# Prevalence of Diabetes on Santa Cruz Island in Galapagos Archipelago

**DOI:** 10.5888/pcd12.150108

**Published:** 2015-06-18

**Authors:** Nicola Tufton, Tahseen Chowdhury

**Affiliations:** Author Affiliation: Tahseen Chowdhury, Royal London Hospital, BartsHealth NHS Trust, London, United Kingdom.

## Abstract

This was an observational study offering a screening program for diabetes in a health clinic in Puerto Ayora town on Santa Cruz Island to determine the prevalence of this disorder and identify those at risk. A 1-month screening program was undertaken. Of 141 patients screened, 85% of men and 83% of women were overweight or obese; 16 (11%) had suspected undiagnosed diabetes and 22 (16%) were at high risk of developing diabetes. This is the first reported study of glucose intolerance prevalence in Galapagos. Urgent education and prevention programs are required to address this public health problem.

## Objective

Diabetes mellitus is an increasing health problem, imposing a public health burden on health systems worldwide (1). The International Diabetes Federation (IDF) reports a prevalence of 6% (1 in 18 adults) in Ecuador and US $562.50 annual health care expenditure per person (2).

The onset of type 2 diabetes can be delayed or prevented; effective management reduces risk of complications. Self-management is the cornerstone of effective care (3). Our study was observational and offered a screening and education program for diabetes in a health clinic in the town of Puerto Ayora to determine prevalence of this disorder and identify those at risk.

## Methods

The Galapagos archipelago, with a population of approximately 23,000 (48.2% female), is 1,000 km (621 miles) off Ecuador’s coast. It consists of 20 islands (5 of which are inhabited) and 250 islets (4). Santa Cruz is the main island and has 61% of the population (approximately 14,000); 78% of the population live in the main town, Puerto Ayora. The age structure of the population on the island consists of 28% aged less than 15 years, 68% aged 15 to 64 years, and 4% aged 65 years or older(5).

Between October 31 and November 29, 2012, a diabetes screening program was offered in the Centro de Salud clinic in Puerto Ayora. The diabetes screening program was advertised weekly on the local television news channel, radio station, and on the Internet. For 1 day (November 14, 2012, National Diabetes Day) screening was offered at a stall set up in the local park by the port.

The International Expert Committee guidelines for diagnosis were used: fasting glucose 126 mg/dL or higher (≥7 mmol/L) for diagnosing diabetes and fasting glucose 100 to 125 mg/dL (5.6–6.9 mmol/L) for diagnosing high-risk individuals with impaired fasting glucose (IFG) (1,6). The committee’s guidelines define high-risk individuals as adults whose body mass index (BMI) is 25 kg/m^2^ or higher with 1 or more of the following risk factors: physical inactivity, first-degree relative with diabetes, high risk race/ethnicity (African American, Latino, Native American, Asian American, Pacific Islander), hypertension 140/90 mm Hg or higher or undergoing therapy for hypertension, and history of cardiovascular disease. We used capillary blood glucose testing on a single finger prick test (7–9).

At presentation to the health clinic, data on the following personal demographics and physical measurements were collected: age, smoking status, personal history of diabetes, weight, height, fasting capillary glucose, and blood pressure. Glucose was tested with Contour test stripes using a Contour (Bayer) glucometer. Weight was measured on a Weigh Beam Eye-Level (Detecto Scale Company) scale (0–175 kg or 0–400 lb).

All patients were provided with education on nutrition and lifestyle changes. Patients with abnormal results were given advice about health concerns and information about improving their diet and increasing physical activity. Appointments were made for repeat testing within 1 to 2 weeks.

## Results

From 5 to 15 patients, including pediatric patients, presented themselves each day to the clinic for various health reasons and were invited to participate in the screening program. Patients were excluded if under the age of 18 years, pregnant, or had not fasted. In total, 141 patients were screened for fasting glucose, and 135 had their weight measured. Six patients refused to have their weight measured or were unable to be weighed (eg, in a wheelchair).

Among those screened, 59.6% were women, and ages ranged from 20 to 78 years (mean, 46 y) (Table 1). Eighty women and 55 men agreed to have their weight measured, and weights ranged from 45 to 120 kg (BMI, 20–44 kg/m^2^) for women and 58 to 120 kg (BMI 23–39 kg/m^2^) for men; 41.8% of men and 45.0% of women were overweight (BMI 25–29.9 kg/m^2^), and 43.6% men and 37.5% of women were obese (BMI ≥30 kg/m^2^). Fasting capillary glucose varied widely, ranging from 51 to 346 mg/dL.

**Table 1:** Demographics of Patients and Fasting Glucose Readings, Puerto Ayora, Galapagos, Ecuador, 2012

Statistic	Age, y	Fasting Glucose, mg/dL (n = 141)	Fasting Glucose, mmol/L (n = 141)	Weight^a^		Body Mass Index^a^	
				Women, kg (n = 80)	Men, kg (n = 55)	Women, kg/m^2^ (n = 80)	Men, kg/m^2^ (n = 141)
Mean	46	110	6.1	71	80	29	29
Range	20–78	51–346	2.83–19.2	45–120	58–120	20–44	23–39
Median	44	96	5.3	65	80	29	28
Mode	40	81	4.5	67	80.5	29	29

a 135 patients had weight measured; 6 patients either refused to be weighed or could not be weighed (eg, in wheelchair, wearing plaster cast).

Of the 141 patients screened, 23 (16.3%) reported that they had previously been told they have diabetes. Of these, 9 were taking at least 1 medication: 6 were taking metformin; 1, sitagliptin; 1, glimepiride; and 2, glibenclamide. No patients were having regular follow-up on the mainland.

Among patients who were not known to have diabetes, 16 had raised fasting glucose levels; 22 patients had IFG (Table 2). Therefore, within this screened population, 11.3% had a likely new diagnosis of diabetes and 15.6% were at high risk of developing diabetes in the future.

**Table 2:** Fasting Capillary Glucose Readings (N = 141), Puerto Ayora, Galapagos, Ecuador, 2012

Capillary Glucose Readings	N (%)	No. (%) With Known Diabetes Mellitus	No. (%) With Suspected New Diagnosis of Diabetes Mellitus^a^	No. (%) Men With Suspected New Diagnosis of Diabetes Mellitus^a^	No. (%) Women With Suspected New Diagnosis of Diabetes Mellitus^a^
≥7.0 mmol/L	30 (21.3)	14 (9.9)	16 (11.3)	5 (31)	11 (69)
5.6–6.9 mmol/L	26 (18.4)	4 (2.8)	22 (15.6)	10 (45)	12 (55)

a Based on only 1 fasting reading.

## Discussion

Our findings suggest substantial numbers of people have undiagnosed, or are at high risk of developing, diabetes. These figures appear higher than those reported by IDF and World Health Organization for Ecuador, especially for the prevalence of obesity (2,10) (Appendix A). Obesity is strongly linked with diabetes risk (11). No published data was found on rates of exercise or hypertension in Galapagos.

Ecuador has close ties to the United States and may be sensitive to US economic, industrial, and cultural influences (12). Galapagos is heavily reliant on tourism, and with an annual population growth of 6.4%, may be at higher risk of increasing obesity (4). Landscape on the islands is not amenable to farming, and farming is carefully managed to protect endemic species. Similar situations have been noted in other island populations, such as the Pacific islands, with increasing prevalence of diabetes and obesity due to rapid population and economic growth and transition to westernization (13). Appendix B shows a comparison of data for diabetes and obesity for Ecuador and other island populations.

Limitations of this study are that it has a small sample size and high dropout rate for repeat checks. Caution should be used when extrapolating outside this population. Bias in sampling may have occurred because only those individuals motivated to seek health advice were screened. Our study may overestimate the prevalence of obesity, as it is unlikely to have data on the more physically active, such as fishermen or cruise ship employees.

Individuals with impaired fasting glucose or impaired glucose tolerance are at high risk for developing diabetes. For populations like that of the Galapagos Islands, which already has multiple risk factors for poor health, there is an urgent need for action. President Rafael Correa suggested introducing a new tax on junk food suppliers to try to address Ecuador’s rapidly rising obesity rates (14).

## Appendix A Prevalence Data for Obesity and Diabetes for Ecuador

**Table:**

Characteristic	World Health Organization (10)		International Diabetes Federation (2)
	Men	Women	
Diabetes	8.7%	9.4%	5.9%
Overweight (body mass index >25 kg/m^2^)	51.8%	60.2%	NA
Obese (body mass index >30 kg/m^2^)	15.7%	28.2%	

Abbreviation: NA, not available.

## Appendix B Prevalence of Diabetes and Obesity Across Various Island Populations

**Table:**

Location	Population	Prevalence of Diabetes, %	Prevalence of Overweight and Obesity (BMI >25 kg/m^2^), %	
			Men	Women
Puerto Ayora (this study)	14,000	11.3	85	82.5
Santa Cruz	14,000	11^a^	NA	NA
Republic of Palau	13,000	18–38.9^b,c^	81.9^d^	81.7^d^
Republic Marshall Islands	32,000	26.5–29.8^c,d^	78.2^d^	82^d^
Federated States of Micronesia	54,000	24.4−36.6^c,d^	71.4^d^	82.5^d^
American Samoa	97,000	47.3^b^	82.6^d^	88.9^d^
Guam	107,000	11–20.1^b,c^	NA	NA
British Virgin Islands	18,000	12.6^c^	NA	NA
Anguilla	9,000	12.6^c^	NA	NA

Abbreviation: BMI, body mass index; NA, information is not available.

^a^ Wilson R. Ponte en forma: an assessment of the health needs of the population of Santa Cruz, Galapagos. August 2009 (unpublished data).

^b^ Hosey et al (13).

^c^ International Diabetes Federation (2).

^d^ World Health Organization (10).
